# Resilience, Anger, and Insomnia in Nurses after the End of the Pandemic Crisis

**DOI:** 10.3390/epidemiologia5040045

**Published:** 2024-10-10

**Authors:** Argyro Pachi, Aspasia Panagiotou, Nikolaos Soultanis, Maria Ivanidou, Maria Manta, Christos Sikaras, Ioannis Ilias, Athanasios Tselebis

**Affiliations:** 1 Psychiatric Department, Sotiria Thoracic Diseases Hospital of Athens, 11527 Athens, Greece; irapah67@gmail.com (A.P.);; 2 Department of Nursing, University of Peloponnese, 22100 Tripoli, Greece; 3 Nursing Department, “Sotiria” General Hospital of Thoracic Diseases, 11527 Athens, Greece; 4 Department of Endocrinology, Hippocration General Hospital, Athens 11527, Greece; iiliasmd@yahoo.com

**Keywords:** insomnia, anger, resilience, post-pandemic, nursing workforce

## Abstract

Introduction: Nurses seem to be persistently experiencing intense psychological repercussions, even after the official conclusion of the COVID-19 pandemic. In this cross-sectional study conducted after the end of the pandemic crisis, from 1 June 2023 to 30 June 2023, we evaluated the levels and explored the associations between anger, insomnia, and resilience among Greek nurses. Methods: A total of 441 nurses participated in an online survey and were invited to state their work experience, gender, and age and to complete the self-report measures of the Dimensions of Anger Reactions-5 (DAR-5), the Athens Insomnia Scale (AIS), and the Brief Resilience Scale (BRS). Results: Overall, 62.1% of the participants presented with positive scores on the AIS, and 41.5% displayed positive values on the DAR-5 scale, whereas 24.9% demonstrated scores indicative of low resilience on the BRS. A regression analysis revealed that 23.5% of the variance in the AIS scores can be attributed to the DAR-5 scores and 3% to the BRS scores. A mediation analysis confirmed the protective role of resilience, contributing as a negative mediator in the DAR-5 and AIS relationship. Conclusions: Screening for insomnia symptoms and anger issues among nurses after the end of the pandemic and implementing appropriate interventions is considered imperative to avoid long-term health consequences.

## 1. Introduction

The American Psychological Association defines resilience as “the process of adapting effectively in the face of adversity, trauma, tragedy, threats or even significant sources of stress” [1]. Essentially, psychological resilience is the ability to endure, recover, and thrive when confronted with stressors and challenging demands. It is a dynamic process that is influenced by various factors including biological, psychological, social, and environmental inputs [2]. The pandemic period was particularly stressful [3,4], especially for healthcare workers, who have experienced additional pressures [5].

Despite past warnings about impending pandemics [6], the coronavirus pandemic took the health systems by surprise, with shortages of personal protective equipment, ventilators, and intensive care beds [7,8]. Public health facilities were often deemed inadequate for the magnitude of the crisis, hospitals in many areas were rapidly overwhelmed, especially during the surge in COVID-19 cases [9,10]. These strains were further intensified for health workers as many of them became sick or were quarantined, leading to increased staff shortages [11,12]. Misinformation and conflicting messages often further complicated the public health response [13,14]. Among health workers, nurses manifested more stress than doctors, making them particularly vulnerable at times when they were most needed [15,16]. Specifically, younger nurses with less clinical experience were more susceptible to adverse mental health outcomes [17,18].

The adverse effects of the pressure exerted by the pandemic on health personnel and especially on nurses were evidenced in numerous studies from the beginning to the end of the pandemic, as the psychological and physical burden on nurses was immense [18]. Sleep disorders, nightmares, and especially insomnia appeared to be a major problem for healthcare workers and especially for nurses [19,20,21]. It should be emphasised here that sleep is a biological necessity for the maintenance of human life comparable to the need for air, water, and food [20,22]. Sleep disturbances are associated with serious physical diseases such as myocardial infarction, hypertension, diabetes [23,24], and major mental disorders such as depression [25,26]. At the same time, an adequate eight hours of sleep at night is associated with a reduced risk of fatigue-related injuries and errors [21].

In the context of adversity during the pandemic, prolonged stress and the perceived threat of COVID-19 triggered feelings of anger among nurses [27]. Anger is one of the basic human emotions [28], involving a complex set of psychological and physiological reactions to injustice, perceived threats, frustration, or even stress [27]. Basic emotions are characterised by the fact that their functioning is essential for adaptation and evolution [29]. Moreover, basic emotions have universal behavioural patterns and most likely an inherent neural substrate [30]. Throughout evolution, the adaptive role of anger in human survival has been related to its involvement in the fight-or-flight response [31]. Although anger has an adaptive role, the exaggerated expression of anger may be maladaptive [32]. Expressions of anger can manifest as early as at six months of age [33,34] and exhibit cross-cultural homogeneity in their basic characteristics [35]. Anger reactions usually peak in early childhood and remit as children socialise and become more capable of regulating emotions [32].

High levels of anger and insomnia were observed in healthcare workers [36] and especially in nurses during the pandemic [27]. The relationship between anger and sleep disorders has been highlighted in several studies, which have suggested that high levels of anger are associated with symptoms of disturbed sleep in adults [37] and with insomnia in adolescents [38]. Recent research evidences an association between sleep quality and the anger expression in nurses [39]. The relationship between the two constructs, namely anger and insomnia, is complex and possibly bidirectional [40,41]. Although it is difficult to disentangle the direction of causation and excluding studies on sleep quality, few studies have examined the effect of insomnia on anger [42,43,44], but research investigating the effect of anger on insomnia is even more limited [45,46,47] and is, in conjunction with resilience and their interrelations, in our opinion non-existent.

The COVID-19 pandemic, which first emerged in early 2020, has become a chronic stressor for healthcare workers and nurses in particular. On 5 May 2023, the World Health Organization announced the “conclusion of the global health emergency caused by the coronavirus disease 2019 (COVID-19)”, effectively putting an end to the pandemic crisis [48,49]. However, experience from previous epidemics has shown that the psychological impact on health professionals can last from several months up to three years after their onset [50]. Four years after the outbreak of the pandemic and with the announcement of its end, one hypothesis that should be explored is whether rates of anger and insomnia have decreased in nursing staff. However, the main purpose of this study was to investigate the protective role of resilience on the relationship between anger and insomnia at the end of the pandemic crisis. The basic assumption was that increased resilience might mitigate the adverse effect of anger on insomnia.

According to this framework, the proposed hypotheses were as follows:

**H1:**  *Anger is positively associated with and predicts insomnia.*

**H2:**  *Resilience is negatively related to and predicts insomnia.*

**H3:**  *Resilience plays a mediating role in the association between anger and insomnia.*

## 2. Materials and Methods

### 2.1. Research Design and Ethical Considerations

To test the above hypotheses we conducted a cross-sectional correlational study. From 1 June 2023 to 31 June 2023, we sent 600 invitations, via email, to registered nurses inviting them to participate in this study by answering the self-report questionnaires. The emails were acquired from professional nurses’ associations. A prerequisite for inclusion in the sample was that the participant had worked for the entire past year in Greece, in any Greek hospital. The survey employed a convenience sampling methodology, since no statistical randomised method was used for the selection of the invited participants. The invitation message stated the purpose of this study and included an anonymous link to access the online survey platform in Google Forms. We also clarified that participation in the survey was voluntary and that we ensured the anonymity of responses. The study sample included 441 individuals (82.1% females and 17.9% males) who agreed to participate (73% response rate). This sample was deemed satisfactory as we had more participants than an adequate sample. Given that the total number of employed nurses in Greece was 27,103 [51], a margin of error of 5%, a confidence level of 95%, and a *p* = 50%, the minimum size was calculated to be 379 employees.

Upon accessing the online survey platform, participants were required to grant online informed consent by answering positively to the question “I agree to participate in this study”, and only with a positive answer could they have access to the questionnaires. This study has been approved by the Clinical Research Ethics Committee of “Sotiria” General Hospital (Number 20649/23). This study adhered to the European Union General Data Protection Regulation (GDPR-2016/679) and was conducted in accordance with the ethical principles as defined by the Declaration of Helsinki.

### 2.2. Measurement Tools

After providing informed consent, participants were asked to state their work experience, gender, and age and subsequently complete the following questionnaires:

(a) The Athens Insomnia Scale (AIS)

To evaluate insomnia we used the Greek version of the Athens Insomnia Scale (AIS), a self-assessment tool designed to quantify sleep disturbances in the last month. The designers used the ICD-10 (International Classification of Diseases 10th revision) criteria for insomnia to generate the scale [52,53]. The AIS is a widely used questionnaire consisting of eight items, such as item 2 (“Awakenings during the night”) and item 3 (“Final awakening earlier than desired”). Each item can be answered on a 4-point Likert scale, where 0 is rated as no problem up to 3 indicating severe difficulties. Thus, the score can range from 0 to 24, where higher scores indicate increased sleep problems [54]. A value of 6 is used as a cut-off point to distinguish healthy individuals from those with insomnia. Values of 15 to 21 indicate moderate insomnia, while values greater than 21 indicate severe insomnia. The internal consistency of the scale is high (Cronbach’s α = 0.89) according to the scale’s authors [52], and in this study, Cronbach’s alpha was equal to 0.86.

(b) Dimensions of Anger Reactions-5 (DAR-5)

The Greek version of the Dimensions of Anger Reactions-5 (DAR-5) scale [55,56] was used to record anger over the past four weeks. The scale consists of five items, measuring individuals’ anger frequency, intensity, duration, antagonism toward others, and interference with social functioning. For example, the first item is “I found myself getting angry at people or situations”. Each item can be rated by the respondent on a 5-point Likert scale ranging from 5 (=always or almost always) to 1 (=never or almost never). Thus, the total score of the questionnaire can range from 5 to 25. High scores indicate a severe experience of anger, while low scores indicate a low experience of anger. The cut-off point is considered to be 12, while internal consistency is usually satisfactory (Cronbach’s α = 0.81) [27,51]. In this study, Cronbach’s alpha was equal to 0.82.

(c) The Brief Resilience Scale (BRS)

We administered the Greek version of the Brief Resilience Scale (BRS) to measure a nurse’s perceived competence in overcoming stress and adversity. The BRS includes six items to which the participant is asked to respond on a five-point Likert scale ranging from strongly disagree (1) to strongly agree (5). For example, the first item is “I tend to bounce back quickly after hard times”. The sum of the scale can yield values from 6 to 30, where low values indicate low resilience. To obtain the final score of the scale, it is necessary to divide the sum by the number of items answered by the respondent. Values lower than 2.99 indicate low resilience and values higher than 4.30 indicate high resilience [57,58]. The internal consistency of the BRS is considered high as reported from previous studies (Cronbach’s α = 0.86) [59]. The Cronbach’s alpha coefficient in this study was α = 083.

### 2.3. Statistical Analysis

Since this study employed a convenience sampling method, it was necessary to examine with the use of chi-square (χ^2^) and *t*-test if this sample was representative in terms of gender, age and years of work as to the general population of nurses in Greece [51,60]. Descriptive statistics served so that continuous variables could be expressed in means and standard deviations. We performed a *t*-test to compare the values of the variables in this study with the values of the same variables during the pandemic from previous studies in the same population. We compared the means of the continuous variables of the present study to gender by *t*-test. In order to estimate the effect size of the *t*-test results, we used Hedges’ g, since in all cases the group sizes we compared were not equal. Values of g close to 0.2 indicate a small effect size, values close to 0.5 a moderate effect size, and values close to 0.8 a high effect size. We tested for correlations between continuous variables with Pearson’s correlation analysis. We verified whether the linear regression assumptions (normality, homoscedasticity, independence, and linear relationship) were satisfied. Linearity was confirmed by visual inspection of PPs and homoscedasticity by visual inspection of the scatter plot of regression standardised residuals and regression standardised predicted values. The Durbin–Watson test was used to detect independence of residuals and the Variance Inflation Factor (VIF) to verify the absence of multicollinearity. The range of Durbin–Watson values is between 0 and 4, where an acceptable range is 1.50–2.50. A value of 2 or close to 2 indicates no autocorrelation. As for the VIF, values less than 1.5 indicate the absence of multicollinearity; however, there are sources that argue that a VIF less than ten may be acceptable. Then, we constructed a regression model to investigate whether the correlated variables were significant predictors of insomnia. Simple mediation analyses were performed using Hayes SPSS Process Macro v4.0 (model 4) [61,62]. In all analyses, statistical significance was set at *p* < 0.05 (two-tailed). IBM SPSS Statistics 23 software was utilised for all statistical analyses (IBM SPSS Statistics for Windows, Version 23.0. Armonk, NY, USA: IBM Corp).

## 3. Results

Initially, to ensure the representativeness of the sample, we compared the characteristics of the sample of this study with the target population, i.e., the total number of working nurses in Greece [60]. No statistical difference was found relating to gender (χ^2^ *p* > 0.05). No statistical difference was found regarding age (*t*-test *p* > 0.05) or years of work (*t*-test *p* > 0.05). In the Athens Insomnia Scale, 62.1% of the sample scored above ≥6, with a percentage of 5.6% presenting moderate and severe insomnia (AIS > 14). In the Dimensions of Anger Reactions-5, a percentage of 41.5% displayed values ≥12. In the Brief Resilience Scale, a percentage of 11.1% exhibited high resilience with values >4.30, while a rate of 24.9% demonstrated low resilience with values <2.99.

In this study, nurse participants scored a mean of 7.32 ± 4.15 in the insomnia scale (Table 1). This value was significantly higher (*t*-test *p* < 0.01) than the value of 5.98 ± 4.24 (Hedges’ g: 0.32) recorded at the beginning of the pandemic in Greek nurses [19] and did not differ from the value of 7.15 ± 4.34 (*t*-test *p* > 0.05) presented in a similar study conducted in the second year of the pandemic at the end of 2021 [63].

Also, nurse participants from this study evidenced higher scores on the DAR-5 scale 11.24 ± 3.85 (Table 1), (*t*-test *p* < 0.01, Hedges’ g: 0.25) compared with the values of 10.31 ± 3.53 recorded in another study at the beginning of the pandemic in Greek nurses using the DAR-5 scale [27].

Meanwhile, the mean resilience score in this study was 3.40 ± 0.78 (Table 1) significantly lower (*t*-test *p* < 0.01, Hedges’ g: 0.28) compared to the value of 3.61 ± 0.80 observed in a study from the initial phase of the pandemic [59].

Investigating the correlations among continuous variables, the expected association between age and work experience was observed, but at the same time, age displayed a weak negative correlation (*p* < 0.05, r = −0.105) with scores on the Athens Insomnia Scale and a weak positive correlation (*p* < 0.01, r = 0.217) with scores on the Brief Resilience Scale (Table 2). Scores on the Athens Insomnia Scale exhibited moderate negative correlations (*p* < 0.01, r = −0.418) with scores on the Brief Resilience Scale and moderate positive correlations (*p* < 0.01, r = 0.485) with the Dimensions of Anger Reactions-5 scale. Also, moderate negative correlations (*p* < 0.01, r = −0.405) were evidenced between scores on the Dimensions of Anger Reactions-5 scale and scores on the Brief Resilience Scale.

Before performing a multiple regression analysis, we examined whether the necessary assumptions were satisfied. We checked linearity by visual inspection of the predicted probability plots (PPs). We examined homoscedasticity through visual inspection of the scatter plot of predicted values standardised by regression and standardised regression residuals. The value of 1.94 (Table 3) in the Durbin–Watson test supports the independence of the residuals, while the values in the Variance Inflation Factor (VIF) analysis exhibited the absence of multicollinearity (Table 3).

To determine which variables best explained the variance in the Athens Insomnia Scale scores, we conducted a multiple regression analysis with the Stepwise method. Insomnia was defined as the dependent variable, while we set gender, work experience, age, and anger as expressed by the Dimensions of Anger Reactions-5 and resilience as recorded by the Brief Resilience Scale as independent variables. The anger scale explained 23.5% of the insomnia scale’s variance, and an additional 5.8% was explained by the resilience scale (Table 3). The remaining variables (gender, age, and years of work experience) were not statistically involved in explaining insomnia.

To investigate whether resilience mediates the anger–insomnia relationship, we used Hayes SPSS Process Macro model 4, based on an analysis of 5000 bootstrap samples. For this purpose, the Brief Resilience Scale was set as the mediator variable, the Athens Insomnia Scale as the outcome variable and the Dimensions of Anger Reactions-5 as a predictor variable. In addition, age and work experience were entered as covariates.

**Figure 1:**
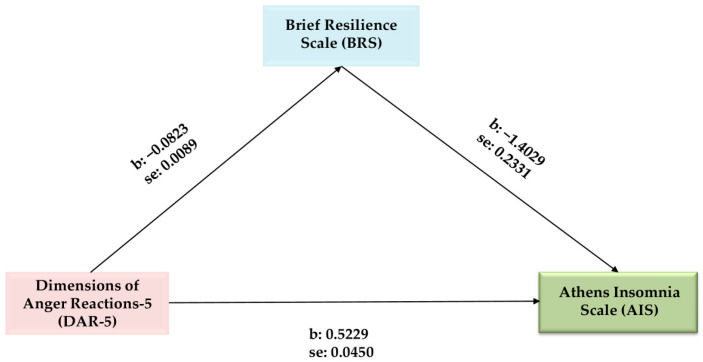
Mediation analysis of Brief Resilience Scale (BRS) on the Dimensions of Anger (DAR-5)/Athens Insomnia Scale (AIS) relationship.

A mediation analysis confirmed our hypothesis that the Brief Resilience Scale negatively mediated the relationship between the Dimensions of Anger Reactions-5 and the Athens Insomnia Scale (Figure 1 and Table 4). The Brief Resilience Scale had a statistically significant but indirect effect [b = 0.1154, 95% CI (0.0712, 0.1669), *p* ≤ 0.01], as in the presence of the mediator (the Brief Resilience Scale), the direct effect of the Dimensions of Anger Reactions-5 on the Athens Insomnia Scale was still statistically significant [b = 0.4074, 95% CI (0.3144, 0.5004), *p* ≤ 0.001). The model explained 22% of the variance in the outcome variable, the Athens Insomnia Scale (Table 4). The variables used as covariates did not display statistically significant relationships. Unstandardized coefficients for the variables with standard errors are illustrated in Figure 1.

## 4. Discussion

The psychological burden placed on nurses had been documented prior to the COVID-19 pandemic [64,65]; however, the unique circumstances of the pandemic dramatically compromised the mental health of nurses. The increased levels of insomnia observed in this study are likely to reflect this deterioration in nurses’ overall mental health. In this study, at the end of the pandemic crisis, a percentage of 62% of participants were symptomatic of insomnia, compared to a rate of 49.7% who were symptomatic in the first year of the pandemic, and not significantly different from the 61.4% in the second year of the pandemic. Studies from around the world confirm that sleep disorders in nurses were a significant problem during the pandemic crisis. In countries where we have comparable data, it seems that in the second year of the pandemic, we have a peak in sleep disorders in nurses. A study in China, using the Athens Insomnia Scale in the first year of the pandemic (March 2020), reports a rate of 41.5% [66], while in a similar study conducted in the second year (March 2021) of the pandemic in China, insomnia symptoms in nurses escalated to 57.3% [67]. In Greece, high rates of insomnia symptoms remained almost invariable until the end of the pandemic crisis [21,63].

The factors responsible for this increase in insomnia rates between the first and the following years of the pandemic could be numerous; first, the course of the pandemic itself and the fatigue it progressively induced to the nursing staff [18], or even the gradual emergence of posttraumatic stress disorder (PTSD) symptoms associated with sleep disorders [67,68]. From a different perspective, we can focus on the protective factors that are beneficial to sleep, such as resilience. Resilience in Greek nurses was reduced at the end of the pandemic crisis compared to measurements in the first year of the pandemic [59]. Another study examining an additional positive factor, family support in Greek nurses, found a similar decline in family support after the first year of the pandemic [20]. It is possible that positive factors were increased in the first year of the pandemic among nurses because of the popularity and even heroism that the profession experienced at the beginning of the pandemic crisis [27,69] that began to fade in the following months. We cannot prove this, but we can assume that the sense of heroism significantly curtailed the sense of anger during the first year of the pandemic.

During the first year of the pandemic, the extremely unfavourable conditions, which have been highlighted in many studies, fully justified the high level of anger experienced by the health personnel. However, we would expect that the normalisation of living conditions would reduce the sense of anger rather than increase it. Prevailing sentiments of anger could be due to the reduced perceived organisational support for nurses [70,71] and increased workplace bullying [72], reflected in lower levels of job satisfaction and higher levels of burnout compared with other healthcare workers [73]. We cannot rule out factors such as trauma or PTSD, also prevalent in veteran soldiers, being responsible for the high sense of anger observed in this study’s sample [45,74,75,76]. There is research suggesting that problematic anger is increased and remains stable across time in repeat military combat recruits compared to first-time service members, as the protective effect of resilience on anger prior to enlistment in the military campaigns is weakened over time [77]. An analogue is viewed in repeated waves of the COVID-19 that probably have a cumulative impact and inflict similar deleterious effects on nurses, undermining resilience and resulting in signs of burnout [78,79,80]. Nurses experienced the COVID-19 pandemic as an ongoing stressor of uncertain duration, and overactivation of the stress system could lower the threshold for problematic anger, effectuating more chronically elevated anger levels [77,81,82]. Whereas in military settings, anger may be more acceptable than other negative emotions [83], in the context of nursing, anger expression may severely compromise the performance and quality of work and adversely impact upon sleep quality [39,84,85]. Existing evidence implicates anger in sleep disorders especially the anger-control deficits [46].

From a neural perspective, insomnia and anger share the same neurobiological substrate associated with abnormal functioning of the amygdala, the ventral anterior cingulate cortex, and the medial prefrontal cortex [43]. As previously stated, anger and sleep probably have a bidirectional relationship, yet in this study, we argued that anger predicts insomnia, and specifically, our study claims that over 23% of the variance in insomnia can be explained by anger. A plausible explanation of how anger contributes to insomnia relies on the cognitive process of rumination of hostile thoughts that are responsible for both maintaining and increasing anger prior to the sleep process [46,86,87,88]. Thus, feelings of anger before sleep onset along with pre-sleep cognitive rumination on an anger provoking stimulus result in physiological and cognitive arousal compromising sleep initiation and maintenance [89,90,91]. However, we should point out that in the literature there is also the opposite view that insomnia and poor sleep are responsible for increased feelings of anger. Previous studies suggest that the quality of sleep the previous night predicted the frequency of anger the next day [92,93] and that the frequency of anger on the current day had no effect on sleep quality [92]. It should be emphasised, though, that the majority of the literature supporting that disturbed sleep is a predictor of anger derives from observations in forensic studies and in studies of psychiatric patients, specifically suggesting that disrupted sleep is a risk factor for impulsive or reactive aggression [94,95].

Hyperarousal and stress dysregulation are implicated in insomnia aetiology [96,97]. Exposure to similar amounts of stress which acts as a precipitating insomnia factor has diverse sleep disturbance consequences among individuals, due to differences in sleep reactivity, which functions both as predisposing and as a perpetuating factor of insomnia [98,99]. Other specific perpetuating factors significantly involved in maintaining persistent insomnia symptoms are sleep effort and pre-sleep cognitive arousal [100]. Insomnia and anger are intimately linked to stress, in the way that the impact of stress on sleep also depends on emotion regulation [101,102]. There is research suggesting that insomnia after stressful life events is more common among individuals with a higher state anger than those without insomnia even after stressful experiences [44]. In this sense, stress-related insomnia is an indicator of sleep reactivity [103] and higher sleep reactivity is related to anger [44]. Sleep reactivity and cognitive-emotional reactivity in response to stress and emotional distress has been associated with the high comorbidity between insomnia and depression [98]. Importantly, REM sleep interferes with emotion regulation through the overnight resolution of emotional distress, a process that is disrupted in insomnia indirectly giving rise to hyperarousal, which further perpetuates insomnia [104].

In this study, resilience displayed a negative association with both anger and insomnia and even mediated their relationship, reducing the adverse effects of anger on sleep. We cannot, however, argue with certainty about the mechanism through which resilience interferes with this relationship, and future studies may be able to provide more evidence. One possible speculation, supported by a recent study, is that resilience acts as a moderator in the relationship between rumination and sleep quality [105]. The study suggests that at high levels of resilience, the impact from daily stressful life events and rumination on sleep quality is expected to be reduced, whereas at low levels of resilience, it is expected to be increased [105].

Previous studies confirm that resilience is compromised among individuals with insomnia compared to good sleepers [106,107,108], reflecting how their low capacity to overcome stress may influence their ability to regulate emotions and arousal, in turn contributing to the maintenance of insomnia [107,108,109]. Differently, emotion dysregulation may impact the relationship between limited capacity to adapt to stress and pre-sleep hyperarousal, creating a vicious cycle that contributes to the chronicity of insomnia [108,110]. Recent research supports that maladaptive emotion regulation strategies undermine the favourable effect of resilience on sleep [111]. In our study, resilience successfully counteracted the effects of anger on insomnia. In accordance, previous studies suggest that resilience has the ability to attenuate feelings of anger and effectively suppresses insomnia [21,59].

The considerable aforementioned findings hold clinical implications that impact not only the occupational performance of nurses but also their overall health [112,113] and underscore the importance of screening for sleep problems and anger issues in nursing personnel after the end of the pandemic. Available effective interventions include evidence-based cognitive behavioural therapies for insomnia [114,115], mindfulness-based cognitive behavioural therapy for anger [116] and educational programmes to enhance resilience [117]. Mounting evidence supporting the use of strategies to improve workers’ self-care demonstrates their protective influence on sleep–wake problems during COVID-19 [118] and for nurses, in particular, interventions that promote self-care, from both personal and professional perspectives, have successfully enhanced resilience [119,120].

This study has several limitations. The shortcomings of self-report measures employed, specifically concerning the retrospective assessment of the variables, could have introduced a more subjective dimension and/or bias. The cross-sectional design of this study would not permit causal inferences, and in this way, the results from this study could be interpreted as indicating that insomnia predicts anger and not vice versa. Future longitudinal investigations could clarify this discrepancy, considering additional reciprocal associations between insomnia and anger. The convenience sampling methodology of this study might have affected the sample’s representativity and the collected data accuracy. Moreover, we did not evaluate stress, other mental health problems, specific sleep-related constructs (i.e., sleep reactivity, chronotypes), personal life-stressors, other work-related factors, personality traits, and/or emotion regulation strategies [20,121,122]. Additionally, this study did not include important positive factors such as the sense of coherence [123,124], social [125] and family support [20,126], or even the sense of religiosity [127] that could act protectively. Also, gender disproportionality prohibited the generalizability of the results to other populations. Finally, excluding nurses without internet access was an important drawback, as was the lack of information about nurses’ working department, shifts at work, rotation, and staff shortages.

## 5. Conclusions

Persistently elevated rates of insomnia and anger are recorded among nursing professionals after the end of the pandemic, whereas self-reported resilience seems to diminish over time. More than six out of ten nurses admit insomnia symptoms; four out of ten nurses experience anger emotions; and one in four nurses exhibits low resilience. Anger exacerbates insomnia and resilience counteracts anger and insomnia. The protective role of resilience is confirmed as it mediates the relationship between anger and insomnia and successfully ameliorates anger and suppresses insomnia. Elevated anger emotions and compromised resilience may contribute to insomnia symptoms perpetuation. In the post-pandemic era, urgent screening for insomnia symptoms among nurses and implementing the necessary interventions is of outmost importance.

## Figures and Tables

**Table 1:** Descriptive statistics of participants.

		Age	Work Experience (in Years)	Dimensions of Anger Reactions-5 (DAR-5)	Athens Insomnia Scale (AIS)	Brief Resilience Scale (BRS)
Male	Mean	46.620 *	20.063	9.848 **	6.051 **	3.599 *
	N	79	79	79	79	79
	Std. Deviation	10.564	11.613	3.146	3.958	0.779
Female	Mean	43.149 *	17.845	11.541 **	7.602 **	3.357 *
	N	362	362	362	362	362
	Std. Deviation	10.838	11.916	3.923	4.139	0.776
Hedges’ g		0.322		0.446	0.378	0.312
Total	Mean	43.771	18.243	11.238	7.324	3.400
	N	441	441	441	441	441
	Std. Deviation	10.859	11.880	3.848	4.146	0.781

* *t*-test *p* < 0.05; ** *t*-test *p* < 0.01.

**Table 2:** Correlations among age, work experience (in years), AIS, DAR-5, and BRS.

		Age	Work Experience (in Years)	AIS	DAR-5
Athens Insomnia Scale (AIS)	r	−0.105 *	−0.077		
	*p*	0.028	0.108		
	N	441	441		
Dimensions of Anger Reactions-5 (DAR-5)	r	−0.056	−0.040	0.485 **	
	*p*	0.238	0.406	0.001	
	N	441	441	441	
The Brief Resilience Scale (BRS)	r	0.217 **	0.185 **	−0.418 **	−0.405 **
	*p*	0.001	0.001	0.001	0.001
	N	441	441	441	441

* Pearson correlations *p* < 0.05, ** Pearson correlations *p* < 0.01. (r: 0.1–03 weak correlation, 0.31–0.7 moderate correlation, 0.71–1 strong correlation.)

**Table 3:** Stepwise multiple regression.

Dependent Variable: Athens Insomnia Scale (AIS)	R Square	R Square Change	Beta	*t*	*p*	VIF	Durbin–Watson
Dimensions of Anger Reactions (DAR-5)	0.235	0.235	0.378	8.609	0.001 *	1.197	1.937
Brief Resilience Scale (BRS)	0.294	0.058	−0.264	−6.019	0.001 *	1.197	

Notes: only statistically significant variables are included; Beta = standardised regression coefficient; * correlations are statistically significant at the *p* < 0.001 level.

**Table 4:** Mediation analysis of the Brief Resilience Scale (BRS) on the Dimensions of Anger Reactions (DAR-5)/Athens Insomnia Scale (AIS) relationship *.

Variables	b	SE	*t*	*p*	95% Confidence Interval	
					LLCI	ULCI
DAR-5 → BRS	−0.0823	0.0089	−9.2896	0.001	−0. 0997	−0.0649
DAR-5 → AIS	0.5229	0.0450	11.6283	0.001	0.4345	0.6112
DAR-5 → BRS → AIS	−1.4029	0.2331	−6.0185	0.001	−1.8610	−0.9448
Effects						
Direct	0.4074	0.0473	8.6092	0.001	0.3144	0.5004
Indirect *	0.1154	0.0244			0.0712	0.1669
Total	0.5229	0.0450	11.6283	0.001	0.4345	0.6112

Notes: * based on 5000 bootstrap samples.

## Data Availability

The data that support the findings of this study are available from the corresponding author (A.T.) upon reasonable request.
